# Perceptions of quality of care in oncological outpatient settings: a qualitative study of healthcare professionals

**DOI:** 10.1136/bmjopen-2025-102950

**Published:** 2025-09-17

**Authors:** Jeanette Kittang, Emma Ohlsson Nevo, Agneta Schröder, Susann Arnell

**Affiliations:** 1 Department of Oncology, Faculty of Medicine and Health, University Healthcare Research Center, Örebro University, Örebro, Sweden; 2 Faculty of Medicine and Health, University Health Care Research Center, Örebro University, Orebro, Sweden; 3 Department of Surgery, Faculty of Medicine and Health, Örebro University, Orebro, Sweden; 4 Department of Health Science, Norges teknisk-naturvitenskapelige universitet, Gjøvik, Norway; 5 Child and Youth Habilitation Centre, Örebro, Sweden

**Keywords:** ONCOLOGY, Quality in health care, QUALITATIVE RESEARCH, Nursing Care, Delivery of Health Care, Integrated

## Abstract

**Abstract:**

**Objectives:**

To explore and describe how healthcare professionals within the oncological outpatient setting perceive quality of care.

**Design:**

A qualitative, descriptive design with a phenomenographic approach was used, with focus group discussions as the means of data collection.

**Setting:**

Primary care in oncological outpatient units in four hospitals in Sweden.

**Participants:**

Through purposive sampling, 20 healthcare professionals entered and completed the study by participating in four focus groups, five participants in each group. Inclusion criteria were assistant nurses, nurses or physicians delivering treatment and care with radiation and/or anticancer drugs in oncological outpatient units. Excluded were healthcare professionals who had worked less than 3 months at the oncological outpatient unit.

**Results:**

Two descriptive categories emerged from the data: ‘The professional’s personal ability for good care’ and ‘The structural conditions for good care’. These categories consist of descriptions of quality of care being perceived as a good meeting with patients, patient participation, continuity, accessibility and care grounded in science.

**Conclusions:**

According to the healthcare professionals, quality of care relies on organisational structures in combination with a professional and personal interaction between the patients and the healthcare professionals. Knowledge about what healthcare professionals believe constitutes quality of care should therefore be highly valuable to policymakers and hospital management.

**STRENGTHS AND LIMITATIONS OF THIS STUDY**

- A strength of the study was that all participants contributed to the discussion, but also that disagreement was present within all four of the groups.
- All focus groups were found to have rich discussions; consensus was found to evolve through discussion, and assertions were challenged in debate. This is considered a strength, as the purpose of focus group discussions is to enrich data through such interactions.
- Variety, as sought by phenomenography, was achieved but with a majority of female participants. This also mirrors reality since healthcare in Sweden is heavily dominated by a female workforce.
- The study lacks perceptions of additional healthcare professions, other than assistant nurses, nurses and physicians. This is by design, as other professions might not exclusively be working only in the outpatient setting or limited to only oncology patients—this can, nonetheless, be seen as a limitation.

## Introduction

 Almost 69 000 people in Sweden receive a cancer diagnosis every year.^1^ The survival rates have though improved due to advances in treatment and earlier detection.^2^ The primary treatment method for solid tumours is surgery, often in combination with oncological treatments with radiation and/or anticancer drugs. Oncological treatments have historically been administered in inpatient wards because of long and complex treatment regimes, infusion-related reactions or heavy side effects.^3^ Over the past two decades, there has been a shift from inpatient to outpatient settings, which has proven beneficial for increasing the patients’ sense of freedom, reducing the clinical inpatient load and reducing costs.^3^ However, beneficial outpatient settings in oncological care might face challenges with limited time and resources.^4^

In the Swedish oncological outpatient setting, patients with curative and palliative oncological treatment intentions are treated in the same room. This requires the healthcare professionals to be educated and prepared to inform patients and start an adjuvant regime as well as give support and comfort in situations where the cancer no longer can be controlled and the oncological treatment is ended—in so-called ‘breakpoint conversations’.^5^ Offering treatment in the oncological outpatient setting is a complex situation, and what quality of care is in this setting is still unclear.

Perceptions of quality of care are found to be important for care utilisation,^6^ and poor quality of care is believed to be a barrier to health coverage.^7^ In Sweden, high-quality care is ensured by law^8^ stating that it is a right of the patient to receive it, and an obligation of the healthcare staff to provide it. However, little is known about quality of care in the oncological outpatient setting and a unanimous definition of quality of care within oncology is still lacking.^9^ Quality of care from the patient’s perspective—both generally^10 11^ and specifically in oncological care^12^,^14^—has been previously described, but the perspectives of professionals also need to be included. Previous research within forensic care^15^ has shown, though, that healthcare professionals’ preferences can differ from what patients find important in care,^16^ hence indicating that the perception of the healthcare professionals is also needed.

When exploring healthcare professionals’ perceptions of quality of care, previous studies have found differing results; Moen *et al*^17^ and Selvin *et al*^16^ discovered that healthcare staff perceive quality of care to be high, while in a study by Roberge *et al*,^18^ the professionals evaluated quality of care less positively than the participating patients.

To our knowledge, no studies have explored the perceptions of healthcare professionals regarding quality of care within the oncological outpatient setting. Therefore, this research project aims to provide a qualitative exploration and description of how healthcare professionals perceive quality of care within the oncological outpatient unit.

## Materials and methods

### Aim of the study

To explore and describe how healthcare professionals within the oncological outpatient setting perceive quality of care.

### Design

A qualitative, descriptive design with a phenomenographic approach was used, with focus group discussions as the means of data collection.

### Phenomenography

As the aim of this study is to focus on how professionals perceive quality of care, phenomenography was chosen as an approach to both data collection and analysis.

Phenomenography^19^ aims at describing the qualitatively different ways that people perceive, understand and think about a phenomenon. Phenomenography focuses on variation and strives for discovering the meaning of phenomena rather than explaining them. This differs from phenomenology, which is more of a philosophical approach when striving to describe the essence of phenomena.^20 21^ Both phenomenography and phenomenology focus on human awareness and the lived experiences.

### Inclusion and exclusion criteria

Inclusion criteria were assistant nurses, nurses or physicians delivering treatment and care with radiation and/or anticancer drugs in oncological outpatient units. Excluded were healthcare professionals who had worked less than 3 months at the oncological outpatient unit. This exclusion criterion was based on a belief that employment shorter than 3 months might risk perceptions from earlier employment elsewhere being expressed.

### Patient and public involvement

Patient or public involvement has not been used in this study.

### Recruitment

Information about the study was presented at professionals’ meetings at four oncological outpatient units at four different hospitals in Sweden. Including these four hospitals contributed to the variation that was sought regarding large cities/rural areas and different county policies. One of the hospitals is a university hospital, and three of the hospitals are regional county hospitals. Those who were interested in participating in the study received written information about the study and the subject of the focus group discussions, along with contact information in case of any questions. A purposive sample was strived for to obtain variation regarding profession, years of experience in the profession, age, sex, workplace and years at the current workplace.

All participants who showed interest in participating were given the opportunity to participate. A total of 20 participants gave their written and informed consent to participate in the study and filled out a short demographic questionnaire (online supplemental file 1), capturing information on their age, sex, profession, family status, years of experience in the profession and years at the current workplace. The participants also received written information about the first author being a doctoral student and a nurse working in the field of oncology. For details on group characteristics, see table 1.

**Table 1:** Characteristics of the participants in the focus groups

	Group 1 n=5	Group 2 n=5	Group 3 n=5	Group 4 n=5
Occupation nurse/assistant nurse/physician	3/1/1	4/1/–	4/1/–	–/–/5
Age range (mean)	28–41 (32.4)	28–55 (42.4)	41–62 (32.6)	37–68 (49.8)
Sex female/male	5/–	4/1	5/–	2/3
Years in occupation range (mean)	3–22 (11)	2–24 (15)	17–43 (25.6)	12–40 (22.4)
Years at current workplace range (mean)	0–22 (6.2)	1–17 (5.8)	1–12 (6.4)	7–40 (19.2)
Length of discussion (hh:mm:ss)	00:40:43	01:01:18	01:08:14	00:48:46
Length of transcript	15 pages	28 pages	22 pages	17 pages

hh:mm:ss, hours:minutes:seconds.

### Data collection

Data were obtained through four focus group discussions, one at each participating hospital, during January and March 2023. Focus group discussions are supposed to lead to deeper dimensions of perceptions to emerge during the discussion because of the active interaction between the participants, making it possible to explore and fertilise different views and opinions.^22^ Data saturation is not central in phenomenography due to the goal of mapping a variety of experiences rather than proceeding until nothing new is presented. The literature recommends between 20 and 30 participants for interviews^23^ and 4–8 groups for focus group discussions.^24^ A sample size of 20 participants divided into four focus groups was, therefore, considered an adequate number of participants both due to phenomenographic tradition and the practicality of four hospitals and one group in each hospital—a group size of five is considered reasonable according to literature on focus groups.^22^ In this study, every focus group met for a single session and there were no dropouts. A semistructured interview guide was used (see table 2).

**Table 2:** Semistructured interview guide

Q1	How do you perceive quality of care within the oncological outpatient unit?
Q2	What is quality of care for you?
Q3	What is quality of care not?
Q4	Can you give an example of when you experienced good quality of care?
Q5	Can you give an example of when you experienced less good quality of care?

The interview guide was prepared in cooperation between all four authors. Probing questions, such as *‘Can you tell me more?’* or *‘Is there someone with a different opinion?’*, were used to initiate further discussions in the groups. The first author moderated three of the discussions and the last author was assistant moderator. The fourth discussion was moderated by the last author and assisted by the third author. During all four of the discussions, the assistant moderator took notes, by hand using pen and paper, on the interaction and flow of conversation within the group, paying special attention to who initiated a new topic, and whether a comment was sent out to the group or directed at a certain participant. All focus group discussions were audio-recorded and later transcribed verbatim by a professional transcriber. Neither transcripts nor findings were returned to participants for comments.

### Data analysis

An inductive analysis of the written transcripts was carried out in four steps following phenomenography by Marton,^20^ using NVivo (V.14).

1. The first author listened to all the audio recordings, ensuring that the transcripts were correct and then read all transcripts multiple times for familiarisation.
2. The first and last author separately identified and marked statements and text sections related to the aim of the study. While reading, the authors also noted words in the margin.
3. Based on similarities and differences, the statements and text sections identified were grouped together into conceptions—focusing on making the conceptions exclusive so that a single statement would not be found to belong in more than one conception.
4. Conceptions were then grouped together, again based on similarities and differences, and descriptive categories emerged. The categories were defined and described based on the conceptions that constitute them. Care was taken to make sure that both borderline and less dominant perceptions were included.

Steps 3 and 4 of the analysis entailed iterative processes in which the categorisations of the conceptions and the definitions of the categories were tested several times. In these steps, all four authors were involved in the analysis process and all four agreed on the result.

The manuscript follows the Consolidated criteria for Reporting Qualitative research checklist.

### Rigour and reflexivity

Credibility within phenomenography is about how well the descriptive categories represent the perceptions of the participants,^19^ presented here through the quotations representing the conceptions and categories. To enable transferability, the authors have provided descriptions of both participant characteristics and the phenomenon to be studied,^25^ making it possible for readers to judge if the result is applicable to other settings.^26^ To achieve dependability, a description of the process of data collection and analysis has been provided.^25^ To enhance trustworthiness, every focus group discussion ended with a summary of what was said in the group. Here, the participants were given the opportunity to provide additional comments and to disagree or correct the verbal summary.

Reflexivity, as critically described by Johnson *et al*,^26^ was practised within the research group through collaboration, conscious conversations and questioning to identify potential assumptions, but also experiences and knowledge.

The research group has different levels of knowledge in different areas, providing valuable knowledge about the organisation of oncological outpatient care, the theoretical study population and quality of care as a phenomenon. The first and last authors are new to the phenomenon and could therefore stay open to the raw data without a preunderstanding of it.

Since the first author works clinically as a nurse in the outpatient unit and the second author is connected to the oncology department as a researcher in one of the hospitals, they did not partake in the focus group discussions held at the specific hospital due to the risk of affecting the participants and the perceptions they choose to share.

All four authors have experience in qualitative research. Three of the authors are nurses and one is a physiotherapist. All four authors identify as female.

## Results

All focus groups were found to have rich discussions; consensus was found to evolve through discussion, and assertions were challenged in debate. See table 3 for examples of this. The analysis of the group dynamics shows that all participants contributed to the focus group discussions, both by introducing perspectives and by reassuring, agreeing or disagreeing with other participants. All participants were actively involved in the focus group discussions, irrespective of age, occupation or experience. Both nurses and physicians disagreed with others occasionally, but the assistant nurses did not express dissenting opinions. In all the groups, every participant contributed by introducing a subject or offering a new input. During the interviews, the participants often returned to the patient perspective and found it difficult to separate their own perspective from the believed wishes of the patients.

**Table 3:** Examples of discussion flow through quotations from transcripts

Example of consensus evolving through discussion.	3: how do you know that you are right in this assessment 4: Mm 2: you don’t, do you. 3: no 2: but it’s a really big difference, today we treat many more at much higher ages who are much sicker and with many more medications. But it’s also erm … 3: and how do you even define what is right to do in such a situation [inaudible] 5: what? 2: we’re talking about how it can also be quality care to for example abstain from treatment and if we do [inaudible] 1: but that I think is really something that … well, we can do more and more, but where is, what is … 5: and more is better, or? 1: yes 5: it isn’t always better. 1: no it really isn’t always better and we have to learn to limit ourselves, so that we can do [inaudible] right to do. It’s really difficult, it’s easier when you don’t have as many things to choose from.
Example of debate where assertions were qualified and challenged.	1: but I think probably we fail sometimes when it comes to continuity, on the nurses’ side as well, I think there are patients who are like, well, am I meeting a new nurse today, or something, it can be like that at times with the treatment, especially if you are here quite often. 3: how do you mean, fail? 1: yeah but well it just feels – 3: you’re not enough? 1: no, you’re not enough, but I think sometimes that it’s something that the patients can feel, ‘What poor continuity this turned out to be’ 2: of nurses? 1: yeah but when you count some of the patients and check their papers, then you can see that there have been new nurses involved in the treatment. 2: although I don’t think that I hear anyone really complaining about that, either, because most of the time they think that ‘Yes, well, now I’ve probably met everyone here, but you’re all so nice’, or, well, but I think it’s more like that. Because I think rather that if it rolls on and the treatment goes on and there’s no problem, well … then … I don’t think it matters that much.

One focus group was a mix of all three participating professions, two groups consisted of nurses and assistant nurses, and one group consisted of physicians only. When presenting quotes, the following abbreviations are used: N for nurses, AN for assistant nurses and P for physicians.

When presenting the results, the term ‘healthcare professionals’ is used when referring to a mix of physicians, nurses and assistant nurses. In cases where participants spoke of a certain occupation only, reference is made to that specific occupation.

The term ‘family member’ is used when referring to a spouse, partner, child, friend or relative.

**Figure 1:**
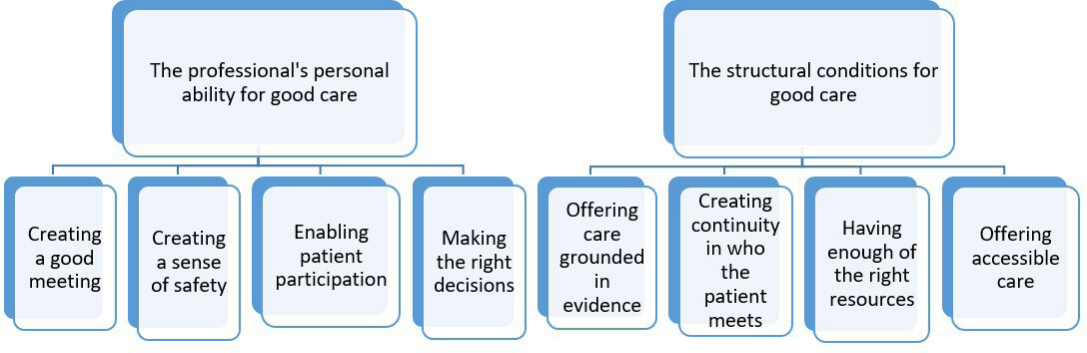
Outcome space.

The outcome space is made up of eight conceptions, forming the two descriptive categories ‘The professional’s personal ability for good care’ and ‘The structural conditions for good care’. According to the phenomenographic approach, these two categories reveal a distinctive understanding; that quality of care is the personal ability of the professionals but also depends on the structural conditions. They are also logically related, since one cannot have one without the other. The categories and conceptions are presented in figure 1 and illustrated by quotations in the text.

### The professional’s personal ability for good care

This descriptive category brings together four conceptions illustrating how quality of care is perceived in terms of the professional’s ability for good care, ranging from individual meetings and participation to involving family and making the right decisions.

#### Creating a good meeting

The participants expressed that it was important to meet the patients in a professional and caring manner. How they meet the patient is crucial, as is being aware of one’s non-verbal presentation, such as body language. The patients are quick to pick up signals and, therefore, the healthcare professionals try to anticipate any misconceptions by, for example, explaining why they will be looking at the clock on the wall, or why they might have to leave the room quickly. Always presenting a calm composure and moving slowly even if stressed, showing interest in what the patient is telling and keeping eye contact are factors that the participants feel contribute to a good meeting.

The professionals addressed the importance of having time, which included personal actions of making time or committing time; having time to see the patient as a person, and meeting this person where they are, listening to their symptoms, but also to listen to important things not related to cancer was experienced as important. Having enough time when meeting with the patients would allow for healthcare professionals to ask the difficult questions. However, time was also described as a structural prerequisite for good care, which is further highlighted later in the results.

When you go in to meet a patient, that you feel you have the time to become involved … That I feel that I have time to, well, to take care of all the things that come up and really see to the patient, that I think is also a very … favourable part of the quality of care here, that you have time for the patient. That it’s not just in and out and do this … the most vital things but you can … give them time, give them the time they need. – N5* *

One specific type of meeting was considered extra important to be a ‘good one’—the meeting where all treatment is ended. Both nurses and physicians described striving for the patient to meet with healthcare professionals they know for this meeting. This was because they knew what had been said earlier and how the patient had reacted—so they could opt for the meeting to be as good as possible. The nurses pointed out that not having enough time to sit down with the patient after such a meeting is perceived as negative for quality of care.

It was … then I know … that no, God, if I had had an opening here, then we would have sat down now, maybe we would have had a cup of coffee together, maybe we really could have just wrapped things up and it would have felt good in my heart when they left. That day was awful, it was my worst day here … – N9* *

Creating good meetings was described as challenging at times as it is not uncommon for them to be met with anger or mistrust. The anger is often a reaction to the situation and is not personal, the healthcare professionals described. They expressed that learning not to take it personally is what enables them to keep their professionalism when patients are angry or yell at them.

Yes, or if it’s an upset relative who you are trying to keep on a good level, that you’re nice anyway and don’t get worked up but that you can … check what is to be done and get back, like that, no but try to be a little of help there, too. Because of course they are worried, too. – AN1

#### Creating a sense of safety

The ability to create an environment with a sense of safety is a crucial part of quality of care, according to the participants. An important element is to deliver the right amount of correct information. The participants described how many patients display concern and uncertainty when coming to the oncological outpatient unit for the first time, and how easily this uncertainty can be reduced through conversation. The participants emphasised that the information needs to be customised to the individual patient. Sometimes, providing the right information is focused mainly on busting myths surrounding cancer treatment.

Well, what people mostly are really scared of is that they are going to be lying there by the toilet and throwing up, and that’s not what they do. – N6

Informing the patients that their treatment proposition has been discussed in teams with multiple specialists prior to the appointment is another way of creating a sense of safety, according to the participants. If the healthcare professionals act in a reassuring way, then it is easier for the patients to feel safe, the participants believed.

Another aspect of creating a sense of safety is contributing to a positive atmosphere. The participants emphasised how both the patients and the healthcare professionals benefit from having an atmosphere where laughter and jokes are included. The participants described how the patients appreciate the smallest of acts and having this positive atmosphere enabled the patient to feel ‘okay’ about returning for another treatment.

And I think that this is a bit of a sign that we, we who work here, that we are quite secure in ourselves, that we are relaxed enough to be able to joke and laugh and then feel—then I think that the patients also feel a little safer than if we were standing there dead serious … – N2

#### Enabling patient participation

The participants emphasised that it is important that patients are made aware of their possibilities to participate in the care. They described that regardless of the prognosis of their disease, all patients should know that they have the right to choose their care and caregiver. The patients should also be made aware that it is not a requirement to accept what is being offered. Patient participation was also described as ‘having the patient on board’, a paraphrase for striving towards the same goal. In situations where the patient declines treatment or does not follow the recommendations given by the healthcare professionals, the participants described feeling content if they believed that the decision was well informed.

If you nonetheless have had a good talk with the patient and then they choose another direction anyway, then I can feel that I’ve done … what I could. That the patient then chooses another thing, fine, yes, as I said, we are different people, we have different opinions and think in different ways. – N6

To enable patient participation, it is important for healthcare professionals to be open and honest with the patient. This was believed to be extra important if an error had been made. Another aspect of being open with the patient was for the nurses to dare to talk about how the patients wish to live their remaining time.

The participants described how patient participation at times might be hindered by language barriers or cultural differences. When healthcare professionals feel there is a risk of not understanding or not making oneself understood, quality of care is impacted negatively.

#### Making the right decisions

An important matter of quality of care is described by the participants as ‘making the right decisions’. For the nurses, this meant the crucial assessment of whether the patient is well enough to receive the planned treatment. At times, making this call becomes more difficult as the patients show different sides to the physicians and the nurses, trying to keep themselves more together when meeting the physician than the nurse. The decision to postpone treatment becomes easier with increased professional experience, the participants emphasised.

I think that if we’re talking about quality of care, then we also make quite a lot of assessments like that of the patient … we can have patients come in who are really not well, who come in to the treatment, where we maybe can go in and say ‘I can’t give you the treatment today because you are—I think you’re not well enough, I have to discuss this with a physician’. – N3

Another difficult decision to make, as described by the participants, is when to end treatment completely. The nurses emphasised that it is good quality of care to know when to end treatment in time. The decision is made by the physicians, but the nurses believed that they themselves should initiate the discussion sooner, as they believed that treatments often go on for too long.

Then there are situations where you feel ‘Well but here you have someone who hasn’t dared to stop the treatment, and you’ve treated them to death’. That I think is poor quality of care. – N10

A challenge regarding making the right decisions is deciding how much influence the patient’s relatives should have. The participants described how strong-willed relatives might affect the decisions too much, overshadowing the patient’s wishes. This was seen as negative for the quality of care within all occupations.

One situation that isn’t that common but when it occurs, then it’s often bad, it’s when you have relatives who really pursue this one line … which isn’t really the patient’s line, perhaps, but then we follow the relatives’ … wishes more because they are so insistent … That can really be not that good a quality of care. – P4

The participants described one invaluable tool while handling decision-making: the support of their colleagues. Being able to discuss patient matters together is believed to be beneficial both for one’s personal development and for the safety of the patient. The participants described an open climate where posing questions between the different professions is welcomed. The open climate also allows you to vent your thoughts and feelings, which is seen as something positive in relation to quality of care.

### The structural conditions for good care

This descriptive category brings together four conceptions illustrating how quality of care is perceived in terms of structural conditions for good care, for example, continuity, accessible care and enough resources.

#### Offering care grounded in evidence

The participants emphasised the importance of care being grounded in science in order to ensure quality of care. The firm base in science was seen as a matter of course by the participants. Keeping up with new knowledge was seen as crucial because of the constant flow of new knowledge regarding new treatment methods and supportive care. Having a work atmosphere where ‘keeping up’ is allowed and encouraged was also emphasised as important.

But I think that here and our staff we have here, that we are really good at keeping up with new things that happen. That we here locally have this culture, I think, because we are good at absorbing information and also communicating it to the patients. – N5

Quality of care was also described as gaining new or deeper knowledge within the field. Both the individual healthcare professionals and the organisation should initiate opportunities to do so. Making sure that the patients were offered all the new drugs and treatment methods that are available and approved in Sweden, and offering them participation in clinical trials, was perceived by the participants as important factors of quality of care.

#### Having continuity in who the patient meets

Continuity was described as an important part of quality of care, both for the participants themselves but also from the patients’ perspective. The healthcare professionals strive for continuity in who the patient meets. They expressed a feeling of failure when continuity is not achieved, since they believed that it is even more important to the patient than to themselves.

And now this was our perspective, but that, the patients’ perspective must become our perspective, too, that’s how it is. And there I think that it is so important that it’s almost impossible to describe, that they … get to meet the same person, it doesn’t have to be exactly the same person, it’s enough that it’s the same people, that it’s like—but not different ones each time. – P2

The participants described positive factors related to continuity, such as gaining both medical and personal knowledge about the patient. Gaining this knowledge, healthcare professionals believed, reduces the risk of missing something important.

It’s also an advantage for us nurses that we get to know the patients so broadly, well we can see from just going to get them from the waiting room that something has happened or now something isn’t quite right. And maybe that doesn’t have anything to do with the treatment, it can be something on an entirely different level, but since we have this continuity, and that also makes it easier for me as a nurse to take care—then they don’t have to tell their whole story because I know their story from the start. So, it’s easier for us in that way, too, to have continuity with the same patient. – N3

Furthermore, the importance of continuity is described by the participants as ‘keeping up’ with the patient, even when they are not at the unit for appointments. Many of the nurses rotate workstations from being ‘in the treatment’ to doing administrative services or responding to the telephone helpline. This rotation makes it possible for them to know what is going on with the patients from many different angles of the ‘care chain’. This knowledge about their patients is also used between colleagues as a way of reporting or seeking advice when treating a patient one has not met earlier.

Yeah, but maybe, you have met this patient more than I have and sort of have a different feeling. I maybe would have given [this treatment] but you have all this other background information that I don’t know, so. – N11

The healthcare professionals also described the possibilities of good quality care when continuity was not achieved. In situations where everything was ‘smooth sailing’ and going according to the treatment plan, continuity in who the patient met mattered less. It could even be positive, since another nurse might discover something that the first one missed. In cases where the personalities of the patient and the healthcare professionals did not match, being able to ‘switch’ patients was seen as a good thing.

#### Having enough of the right resources

The right resources were described by the participants both in terms of physical resources and in terms of time. Having the right physical resources, such as sexologists, welfare officers, nurse assistants or medical secretaries, and enough of them, was mentioned as important for quality of care. This was especially visible in the units experiencing a shortage or lack of specific resources.

Lack of nurses and other groups … if they are not doing so well, then we are not doing so well either, that it becomes more of a mess in general. More things go wrong or more questions and … it’s an uncertainty that is noticeable, anyway … so all groups have to be doing well enough to be able to offer good quality of care. – P6

The participants described having enough time for one’s work tasks as a crucial factor for quality of care, and not having enough time to support colleagues was perceived negatively.

And with lack of time, which means that we can’t make decisions within the time we would have liked to have or asked for but make quick decisions and then it’s easy to make mistakes. – P6

Interruptions were seen as harmful, since they created a vicious cycle of missing important things and then being interrupted again to correct what had been missed. The effect on quality of care was highlighted by the participants by describing interruptions taking place in the middle of meeting with a patient, resulting in a less good meeting and risking causing stress or concern within the patient.

#### Offering accessible care

An important factor for quality of care, as described by the participants, is being able to offer care that the patients can access. Accessibility is described as a complex factor since every patient expects different levels of accessibility from the healthcare. Healthcare professionals described various ways of providing access: via telephone, digital question boxes and local technical support.

Accessibility also concerns physical access and having the possibility of care in the patient’s home county. Due to centralisation, many patients are forced to travel for hours to receive their cancer treatment. While acknowledging the benefits of centralisation, the participants emphasised that it causes inequity and negatively impacts the quality of care.

Having access to care within a reasonable timeframe was emphasised by the participants as important for quality of care. Having to wait for appointments is believed by healthcare professionals to cause concern in the patients. The complexity of waiting time was described by the participants when there was no medical reason to indicate hasty actions.

But it’s always been like that that we don’t think there’s any need to rush … really, from a medical viewpoint, but the patients can experience that it is urgent anyway. – P4

Another aspect of accessibility is the collaboration between various healthcare units. Cooperation was described in positive terms regarding quality of care, highlighting the importance of teams working closely together and being aware of everyone’s different roles. Having routines for collaboration and knowledge of the entire ‘care chain’ is seen as crucial for quality of care.

## Discussion

The findings show that quality of care from the healthcare professionals’ perspective can be described through two descriptive categories: ‘The professional’s personal ability for good care’ and ‘The structural conditions for good care’. This indicates that quality of care can be perceived as something with a positive value and a strong commitment within the oncological outpatient setting, as long as necessary resources for providing quality of care are present.

In the present study, continuity, accessible care based on evidence and enough resources were structural conditions linked to perceived quality of care. The importance of having collaboration within and between professions to achieve quality of care was highlighted by the healthcare professionals. Collaboration is a cornerstone in healthcare^27^ and is dependent on adequate staffing,^28^ organisational leadership and time.^29^ In the present study, adequate staffing was described in terms of having the right resources, time for the patient, and for one’s duties, and was believed to contribute to quality of care. Inadequate staffing has been connected to patient safety outcomes, emotional exhaustion^30^ and burnout.^31^ The participants in the present study especially described negative feelings when not being able to offer their patients quality care. This is important given that engaged and satisfied healthcare professionals are positively correlated with patient safety and quality of care.^32^ Continuing from this, the participants in the present study emphasised that not having enough time to consult with colleagues and having to make difficult decisions too quickly were key issues, highlighting the interconnectedness of collaboration, staffing and time, all of which influence one another. In terms of quality of care, this interconnectedness is crucial, as slower decisions tend to be more accurate.^33^

When the healthcare providers described how they perceive quality of care, they often returned to the patient perspective. It seemed difficult to separate their own perspective from the believed wishes of the patients. This indicates that professionals are committed and what is important to the patient will likely be important to the healthcare professionals. Similar findings have previously been seen in the psychiatric setting, where the staff described quality of care in terms of the patients’ health and well-being.^34^

Quality of care in the oncological outpatient setting has previously been described from the patients’ perspective, as a positively charged phenomenon, and it is considered normative, ie,how the care should be.^12^ This differs from the healthcare professionals’ perspective in the present study where the concept is neutral and dependent on the staff rather than normative. Another difference between patients’ and healthcare professionals’ perceptions is the involvement of family. While the patient study^12^ found family involvement to be very important, there were very few statements in the present study that mentioned family involvement, and when doing so, healthcare professionals only focused on how to deal with upset family members or how much influence the family should have on medical decisions. If it was because of a negative attitude towards family involvement, this would differ from previous research where healthcare professionals have reported positive attitudes^35^ and see family as a resource in clinical work.^17^ The perception of family involvement as challenging, on the other hand, is supported by many previous studies.^36 37^ A common challenge, as also seen in the present study, was the conflicting treatment wishes when the family wanted one thing and the patient wanted another,^36^ or when the patient was being pressured by their family to continue with therapy.^37^

### Strengths and limitations

A strength of the study was that all participants contributed to the discussion, but also that disagreement was present within all four of the groups. Since the composition of the groups is crucial in a focus group study,^22^ and since phenomenography strives for variation, it was desirable to have variation within the focus groups. The composition varied between the groups by design. One of the groups was a mix of all three professions: nurses, assistant nurses and physicians. Two groups consisted of nurses and nurse assistants, and one group consisted solely of physicians. The choice not to include physicians in all groups was based on the hierarchical structure within healthcare, where physicians are placed above nurses and assistant nurses by the level of medical knowledge. It was, therefore, hypothesised that including physicians in every group might hinder the other professions from airing alternative opinions. The authors cannot determine whether any opinions were left out, but the analysis indicates that the participants felt secure enough to share their perceptions.

The professions chosen for the study are the ones working closest to the patient in the oncological treatment care. Even though professions such as dieticians and welfare officers are linked to the outpatient setting, they are not exclusively working only in the outpatient setting or limited to only oncology patients—there would have been a risk that perceptions of quality of care might be influenced by perceptions in more general care. Nevertheless, the results, therefore, lack perceptions of additional healthcare professions which can be seen as a limitation of the study.

### Clinical implications and future research

Knowledge about what healthcare professionals believe constitutes quality of care should be highly valuable to policymakers and hospital management, because even though the patient perspective is essential to quality of care,^38^ the present study wishes to highlight the importance of incorporating the professionals’ perspective as well. Quality of care cannot be achieved without healthcare staff, and the literature indicates that the well-being of the healthcare staff is connected to quality of care provided.

The findings of this study will be used in the development of an assessment instrument to evaluate quality of care in oncological outpatient settings. As a planned next step, our research team will focus on designing and validating this instrument to enhance clinical practice and patient outcomes.

## Conclusions

According to the healthcare professionals, quality of care within the oncological outpatient setting relies on organisational structures in combination with professional and personal interactions. Healthcare professionals believe that there are certain things that are prerequisites, which the organisation needs to provide, for them to be able to provide good quality of care. The authors believe that both the quality of care and the work environment could benefit from healthcare professionals voicing these prerequisites more strongly and confidently in the future. We also see the necessity of management creating a culture where voicing concerns is regarded as positive.

## Supplementary material

10.1136/bmjopen-2025-102950 online supplemental file 1

## Data Availability

Data are available on reasonable request.
