# Supplementary material for: Perceptions of quality of care in oncological outpatient settings: a qualitative study of healthcare professionals
Source: BMJ Open. 2025 Sep 17;15(9):e102950. doi: 10.1136/bmjopen-2025-102950 (PMC12458673; doi:10.1136/bmjopen-2025-102950)
Supplement: online supplemental file 1 [file bmjopen-15-9-s001.docx]

**Supplementary file** Demographic questionnaire

Year of birth ______________________________________

Gender Female/Male/Other/Do not wish to answer

Place of residence _______________________________________

Civil status Living with someone/Living alone

Country of birth Born in Sweden/Born outside of Sweden

Education Level Elementary/Upper secondary/University

Profession Nurse assistant/Nurse/Physician

Number of years in the profession _________________________________

Workplace _______________________________________

Number of years in current workplace ______________________________
