# Supplementary material for: Perceptions of quality of care in oncological outpatient settings: a qualitative study of healthcare professionals
Source: BMJ Open. 2025 Sep 17;15(9):e102950. doi: 10.1136/bmjopen-2025-102950 (PMC12458673; doi:10.1136/bmjopen-2025-102950)
Supplement: Supplementary file 2 [file bmjopen-2025-102950.reviewer_comments.pdf]

PEER REVIEW HISTORY

BMJ Open publishes all reviews undertaken for accepted manuscripts. Reviewers are asked to complete a checklist review form and are provided with free text boxes to elaborate on their assessment. These free text comments are reproduced below.

ARTICLE DETAILS

Title (Provisional)

Perceptions of Quality of Care in Oncological Outpatient Settings: A Qualitative Study of Healthcare Professionals.

Authors

Kittang, Jeanette; Ohlsson Nevo, Emma; Schrøder, Agneta; Arnell, Susann

---

VERSION 1 - REVIEW

**Reviewer**	**1**
**Name**	**Lamprell, Klay**
**Affiliation**	**Macquarie University, Australian Institute of Health Innovation**
**Date**	**23-Jun-2025**
**COI**	**None**

This is an important topic, the study is well-designed and the findings are interesting and make a significant contribution. There are a number of issues that should be addressed to make this a paper of substantial value.

Method

2.3 I would have liked to see an explanation of di8erence between phenomenography & phenomenology to highlight that phenomenology explores the essence of individual experiences, while phenomenography examines the di8erent ways people understand the same phenomenon.

2.4 I would have liked to see an explanation of why exclusion for employment less than 3 months.

2.6 Why these four hospitals in particular? If aiming to achieve variation in profession, years of experience, age, gender, and years at current workplace, did these four hospitals provide that variation?

2.7 Would have liked to see an explanation of the data saturation policy e.g. that data saturation isn’t central in phenomenography because the focus is on mapping the

range of di8erent experiences, not collecting data until repetition occurs, rather than relating it to methodological 'tradition' Additionally, there would be value in a justification for why a sample size around 20 participants was considered adequate.

2.8 Why 'inspired by'? Does this mean the authors have adapted a model by Marton? OR is it referring to the fact that Marton doesn't explicitly number "four steps" in his entry, but his method is widely interpreted as a four-stage analytic process?

Results

3.1.1.1. Time is noted as a structural element also – it should be di8erentiated here from the personal action of making time or committing time.

Discussion

There are structural and content-related problems with the Discussion. - In terms of content, the opening paragraph discusses 'facilitators and obstacles' as a key finding but this is not shown in the Results so it cannot be considered a key finding. Instead, the Discussion could propose a 'facilitators and obstacles' framework for future understanding of the findings, and then reference this framework to its foundation principles which can be found in implementation science. Nor do the Results show that healthcare sta8 often returned to the patient perspective found it di8icult to separate their own perspective from the believed wishes of the patients. The Discussion should relate only to what is shown in the Results so either discard this or show it explicitly in the Results.   The content in the paragraph on quality of care being previously described from the patients' perspective as normative and positive needs to be more e8iciently composed and provide clarification as to what is being communicated. - In terms of structure, the Strengths & Limitations text starts in the second paragraph of the Discussion when it should be at the end of the Discussion section; additionally it is too long and adds little value to the Discussion. It runs from P18, Line 32 through to P19 Line 30.

Conclusion i

Thee Conclusion is weak. Either in the Discussion or here, there needs to be more focus on the translation of findings into policy and practice.

Overall Language

Figure 1 there is a spelling mistake in 'enabling'; Throughout the results some descriptions of the themes are long-winded and could be more e8iciently written. In the Discussion, check for grammar, e.g. *"the present study wish…"*

Visualisation of findings: Would see value in either extending Figure 1 to include sub-themes or providing a new figure/graphic showing sub-themes. For example, under 'creating a sense of safety' show the sub-themes of myth-busting, MDT assurance, and

positive environment.

Referencing

The manuscript would be improved by the inclusion of more recent references in relation to quality of care, to ensure that the most recent literature on the topic has been covered.

**Reviewer**	**2**
**Name**	**Pini, Simon**
**Affiliation**	**University of Leeds, Psychological and Social Medicine**
**Date**	**26-Jun-2025**
**COI**	**None**

Thank you for the invitation to review this paper. It is an interesting and worthwhile area of research. Overall I think this paper is very well written and presented, so I congratulate the authors on the efforts made with this manuscript.

My comments/questions are fairly minor and I have broken them down by section:

Overall:

My main comment about the paper is that the instrument develop feels like an “afterthought” in this paper, but is perhaps a key reason why this piece of work was conducted? I would consider adding details of the planned instrument development in more detail throughout the paper. It should be an explicit aim, with an explicit plan for how the results will inform the development of this instrument. The plan can be brief, as I know this is not the main focus of the paper, but a little detail about how the team plan to use the findings to develop the instrument would be a good addition. Otherwise, I think you could remove the very brief mentions of the instrument development and leave the paper as a stand alone qualitative study. But my preference would be for a little more detail about this proposed instrument.

Abstract:

Is the design cross-sectional? It seems so, so I would add this.

The “instrument” is only mentioned in the objectives and conclusions. Is there room to say anything about how the findings were/will be shaped towards the development of the instrument. You mention validating the instrument, but the abstract suggests the instrument is not created yet, so would it be more appropriate to say you will develop the instrument?

Introduction:

Can there be a little more detail added after "ensured by law"? What does the law say?

The final section is in a larger typeface.

Methods:

The aim does not include developing the instrument. I would add that as an additional aim.

Add that the study was cross-sectional.

Can you explain why there was no PPI in the study? I know it is focussed on healthcare professionals, but perhaps you can just explain this rationale clearly in the paper with an additional sentence or two.

Do you have the demographic questionnaire in the supplementary files? I think it would be good to see these.

How were the notes made by the assistant moderator in the focus groups used? Perhaps mention that here in the method, although I know you mention it in the results.

Results:

I think these are clear and well presented with good use of participant quotes.

Discussion:

Again I think this is well written and reflects well on the results, literature and wider context of the topic area. Are there specific practical things that healthcare professionals could do differently as a result of this study, or how they could think differently about how they work with patients?

VERSION 1 - AUTHOR RESPONSE

Reviewer 1

Method

2.3 I would have liked to see an explanation of difference between phenomenography & phenomenology to highlight that phenomenology explores the essence of individual experiences, while phenomenography examines the different ways people understand the same phenomenon.

Authors' response: We are grateful for this suggestion. To clarify the difference between phenomenography & phenomenology the manuscript has been revised with the following, lines 22-24, on page 4: This differs from phenomenology which is more of a philosophical approach when striving to describe the essence of phenomena (20, 21). Both phenomenography and phenomenology focus on human awareness and the lived experiences.

2.4 I would have liked to see an explanation of why exclusion for employment less than 3 months.

Authors' response: Thank you for bringing to our attention the lack of explanation regarding the exclusion criteria. The manuscript has been revised with the following, lines 2-3, on page 5: This exclusion criterion was based on a belief that employment shorter than 3 months might risk for perceptions from earlier employment elsewhere to be expressed.

2.6 Why these four hospitals in particular? If aiming to achieve variation in profession, years of experience, age, gender, and years at current workplace, did these four hospitals provide that variation?

Authors' response: We appreciate this helpful observation and have now added a description of how the four hospitals added to the variation. The manuscript has been revised with the following, lines 10-12, on page 5: Including these four hospitals contributed to the variation that was sought regarding urban cities/rural areas and different county policies.

2.7 Would have liked to see an explanation of the data saturation policy e.g. that data saturation isn't central in phenomenography because the focus is on mapping the range of different experiences, not collecting data until repetition occurs, rather than relating it to methodological 'tradition' Additionally, there would be value in a justification for why a sample size around 20 participants was considered adequate.

Authors' response: The manuscript has been revised with the following, lines 3-10, on page 6: Data saturation is not central in phenomenography due to the goal of mapping a variety of experiences rather than proceeding until nothing new is presented. The literature recommends between 20 and 30 participants for interviews (23) and 4-8 groups for focus group discussions (24). A sample size around 20 participants divided into four focus groups, was therefore considered an adequate number of participants both due to phenomenographic tradition and the practicality of four hospitals and one group in each hospital – a group size of five is considered reasonable according to literature on focus groups (22).

2.8 Why 'inspired by'? Does this mean the authors have adapted a model by Marton? OR is it referring to the fact that Marton doesn't explicitly number "four steps" in his entry, but his method is widely interpreted as a four-stage analytic process?

Authors' response: The authors apologize for the poor choice of wording, leading to misunderstanding. The manuscript has been revised with the following, lines 27-28, on page 6: An inductive analysis of the written transcripts was carried out in four steps following phenomenography by Marton (20), using NVivo (version 14).

Results

3.1.1.1. Time is noted as a structural element also – it should be differentiated here from the personal action of making time or committing time.

Authors' response: Thank you for bringing to our attention that time as a structural element needs to be separated. The manuscript has been revised with the following, lines 10-13, and 15-16, on page 10: The professionals addressed the importance of having time, which included

personal actions of making time or committing time; Having time to see the patient as a person, and meeting this person where they are, listening to their symptoms, but also to listen to important things not related to cancer was experienced as important. Having enough time when meeting with the patients would allow for healthcare professionals to ask the difficult questions. However, time was also described as a structural prerequisite for good care, which is further highlighted later in the results.

Discussion

- There are structural and content-related problems with the Discussion.

In terms of content, the opening paragraph discusses 'facilitators and obstacles' as a key finding but this is not shown in the Results so it cannot be considered a key finding. Instead, the Discussion could propose a 'facilitators and obstacles' framework for future understanding of the findings and then reference this framework to its foundation principles which can be found in implementation science.

Authors' response: Thank you for your suggestion about the framework and for pointing out the ambiguity in the first part of the discussion. We have revised and chosen to clarify the most important results, lines 13-18, on page 17.

- Nor do the Results show that healthcare staff often returned to the patient perspective found it difficult to separate their own perspective from the believed wishes of the patients. The Discussion should relate only to what is shown in the Results so either discard this or show it explicitly in the Results.

Authors' response: Thank you for bringing this error to our attention. The manuscript has been revised, in the result section, with the following, lines 1-2, on page 9: During the interviews, the participants often returned to the patient perspective and found it difficult to separate their own perspective from the believed wishes of the patients.

- The content in the paragraph on quality of care being previously described from the patients' perspective as normative and positive needs to be more efficiently composed and provide clarification as to what is being communicated.

Authors' response: We are grateful for this suggestion. The manuscript has been revised with the following, lines 11-15, on page 18: Quality of care in the oncological outpatient setting has previously been described from the patients' perspective, as a positively charged phenomena, and it is considered normative, i.e how the care should be (12). This differs from the health care staffs' perspective in the present study where the concept is neutral and dependent on the staff rather than normative.

- In terms of structure, the Strengths & Limitations text starts in the second paragraph of the Discussion when it should be at the end of the Discussion section; additionally it is too long and adds little value to the Discussion. It runs from P18, Line 32 through to P19 Line 30.

Authors' response: The authors has revised the Strengths & Limitations section, moving it to the end of the discussion and shortened the section.

Conclusion

- The Conclusion is weak. Either in the Discussion or here, there needs to be more focus on the translation of findings into policy and practice.

Authors' response: The manuscript has been revised with the following, lines 6-9, on page 20: The authors believe that both the quality of care and the work environment could benefit from healthcare staff voicing these prerequisites more strongly and confident in the future. We also see the necessity of management creating a culture where voicing concerns is regarded as positive.

Overall Language

- Figure 1 there is a spelling mistake in 'enabling'.

Authors' response: Thank you for bringing this error to our attention. The spelling has now been corrected.

- Throughout the results some descriptions of the themes are long-winded and could be more efficiently written.

Authors' response: Thank you for bringing this to our attention. The authors have revised some of the long-winded descriptions in the result section.

Page 10, lines 23-26.

Page 12, lines 6-9.

Page 12, lines 22-24.

Page 14, lines 25-28.

Page 15, lines 14-17.

Page 15, lines 24-26.

- In the Discussion, check for grammar, e.g. "the present study wish…"

Authors' response: The authors has revised the discussion section and corrected grammar accordingly.

- Visualisation of findings: Would see value in either extending Figure 1 to include subthemes or providing a new figure/graphic showing sub-themes. For example, under 'creating a sense of safety' show the sub-themes of myth-busting, MDT assurance, and positive environment.

Authors' response: Thank you for this comment. The authors have considered this suggestion, and even though we see the benefits, we find it to not agree with the phenomenographic outcome space according to Marton and we therefore wish to not apply to this suggestion.

Referencing

- The manuscript would be improved by the inclusion of more recent references in relation to quality of care, to ensure that the most recent literature on the topic has been covered.

Authors' response: Thank you for this comment. The authors have revised the background with more recent references.

Reviewer 2

Overall:

- My main comment about the paper is that the instrument develop feels like an "afterthought" in this paper, but is perhaps a key reason why this piece of work was conducted? I would consider adding details of the planned instrument development in more detail throughout the paper. It should be an explicit aim, with an explicit plan for how the results will inform the development of this instrument. The plan can be brief, as I know this is not the main focus of the paper, but a little detail about how the team plan to use the findings to develop the instrument would be a good addition. Otherwise, I think you could remove the very brief mentions of the instrument development and leave the paper as a stand alone qualitative study. But my preference would be for a little more detail about this proposed instrument.

Authors' response: Thank you for this comment, making us aware of the confusion we have caused. The result from this study will be used further on, in the development of an instrument – but it is not the key reason for the present study. The manuscript has therefor been revised, and the mentioning of the instrument development has been removed.

Abstract:

- Is the design cross-sectional? It seems so, so I would add this.

Authors' response: Thank you for this comment. The authors believe cross-sectional to be a design more used in quantitative research. Hence, we do not wish to add the design to the manuscript.

- The "instrument" is only mentioned in the objectives and conclusions. Is there room to say anything about how the findings were/will be shaped towards the development of the instrument. You mention validating the instrument, but the abstract suggests the instrument is not created yet, so would it be more appropriate to say you will develop the instrument?

Authors' response: Thank you for this comment. As mentioned above, the manuscript has been revised, and the mentioning of the instrument development has been removed.

Introduction:

- Can there be a little more detail added after "ensured by law"? What does the law say?

Authors' response: The manuscript has been revised with the following, lines 20-22, on page 3: In Sweden, high quality care is ensured by law (8) stating that it is a right of the patient to receive it, and an obligation of the healthcare staff to provide it.

- The final section is in a larger typeface.

Authors' response: Thank you for bringing this error to our attention – we have revised the section to the correct typeface.

Methods:

- The aim does not include developing the instrument. I would add that as an additional aim.

Authors' response: Thank you for this comment. As mentioned above, the manuscript has been revised, and the mentioning of the instrument development has been removed.

- Add that the study was cross-sectional.

Authors' response: Thank you for this comment. The authors believe cross-sectional to be a design more used in quantitative research. Hence, we do not wish to add the design to the manuscript.

- Can you explain why there was no PPI in the study? I know it is focussed on healthcare professionals, but perhaps you can just explain this rationale clearly in the paper with an additional sentence or two.

Authors' response: Thank you for this comment. PPI was not included in this study as the focus was specifically on the experiences and perspectives of healthcare professionals. Given the scope, patient or public involvement was not considered directly relevant. The authors do not believe that the manuscript will benefit from additional elaboration.

- Do you have the demographic questionnaire in the supplementary files? I think it would be good to see these.

Authors' response: We are grateful for this suggestion. The authors have added the demographic questionnaire to the supplementary files.

- How were the notes made by the assistant moderator in the focus groups used? Perhaps mention that here in the method, although I know you mention it in the results.

Authors' response: The authors believe that this information is already provided in the method section (2.7), lines 18-22, on page 6: "During all four of the discussions, the assistant moderator took notes on the interaction and flow of conversation within the group, paying special attention to who initiated a new topic, and whether a comment was sent out to the group or directed at a certain participant."

The manuscript has been revised with the following for clarity: ...took notes by hand using pen and paper, on the interaction.

Results:

- I think these are clear and well presented with good use of participant quotes.

Authors' response: Thank you for your kind words.

Discussion:

- Again, I think this is well written and reflects well on the results, literature and wider context of the topic area. Are there specific practical things that healthcare professionals could do differently as a result of this study, or how they could think differently about how they work with patients?

Authors' response: Again, Thank you for your kind words. The manuscript has been revised with the following, lines 6-9, on page 20: The authors believe that both the quality of care and the work environment could benefit from healthcare staff voicing these prerequisites more strongly and confident in the future. We also see the necessity of management creating a culture where voicing concerns is regarded as positive.

VERSION 2 - REVIEW

**Reviewer**	**1**
**Name**	**Lamprell, Klay**
**Affiliation**	**Macquarie University, Australian Institute of Health Innovation**
**Date**	**04-Aug-2025**
**COI**	

Thank you for your thoughtful and thorough revisions. You have addressed all of the points raised and clearly made every effort to strengthen the manuscript. I believe the manuscript is now more robust and impactful for its intended readership.
